# Supplementary material for: Pyruvate Dehydrogenase Kinase 1 inhibition mediated oxidative phosphorylation enhancement in cartilage promotes osteoarthritis progression
Source: BMC Musculoskelet Disord. 2023 Jul 20;24:597. doi: 10.1186/s12891-023-06585-6 (PMC10357736; doi:10.1186/s12891-023-06585-6)
Supplement: Supplementary file 1 — Additional file 1. [file 12891_2023_6585_MOESM1_ESM.zip › BP.Gsea.1653623667859/GOBP_REGULATION_OF_MRNA_METABOLIC_PROCESS.html]

Details for gene set GOBP\_REGULATION\_OF\_MRNA\_METABOLIC\_PROCESS[GSEA]

|  || Dataset | 55457\_collapsed\_to\_symbols.cls.cls #OA\_versus\_normal.cls.cls #OA\_versus\_normal\_repos |
| Phenotype | cls.cls#OA\_versus\_normal\_repos |
| Upregulated in class | normal |
| GeneSet | GOBP\_REGULATION\_OF\_MRNA\_METABOLIC\_PROCESS |
| Enrichment Score (ES) | -0.41024947 |
| Normalized Enrichment Score (NES) | -1.9920704 |
| Nominal p-value | 0.0 |
| FDR q-value | 0.034675125 |
| FWER p-Value | 0.311 |
Table: GSEA Results Summary

  

Fig 1: Enrichment plot: GOBP\_REGULATION\_OF\_MRNA\_METABOLIC\_PROCESS      
 Profile of the Running ES Score & Positions of GeneSet Members on the Rank Ordered List

  

| SYMBOL | TITLE | RANK IN GENE LIST | RANK METRIC SCORE | RUNNING ES | CORE ENRICHMENT || 1 | POLR2G | RNA polymerase II subunit G [Source:HGNC Symbol;Acc:HGNC:9194] | 183 | 0.830 | -0.0044 | No |
| 2 | PAN2 | poly(A) specific ribonuclease subunit PAN2 [Source:HGNC Symbol;Acc:HGNC:20074] | 190 | 0.828 | 0.0052 | No |
| 3 | FAM172A | family with sequence similarity 172 member A [Source:HGNC Symbol;Acc:HGNC:25365] | 251 | 0.785 | 0.0100 | No |
| 4 | SRPK3 | SRSF protein kinase 3 [Source:HGNC Symbol;Acc:HGNC:11402] | 329 | 0.734 | 0.0128 | No |
| 5 | MLH1 | mutL homolog 1 [Source:HGNC Symbol;Acc:HGNC:7127] | 497 | 0.660 | 0.0076 | No |
| 6 | SRSF9 | serine and arginine rich splicing factor 9 [Source:HGNC Symbol;Acc:HGNC:10791] | 882 | 0.548 | -0.0161 | No |
| 7 | SAP18 | Sin3A associated protein 18 [Source:HGNC Symbol;Acc:HGNC:10530] | 1099 | 0.496 | -0.0271 | No |
| 8 | NBAS | NBAS subunit of NRZ tethering complex [Source:HGNC Symbol;Acc:HGNC:15625] | 1132 | 0.490 | -0.0237 | No |
| 9 | NELFE | negative elongation factor complex member E [Source:HGNC Symbol;Acc:HGNC:13974] | 1135 | 0.489 | -0.0179 | No |
| 10 | MBNL3 | muscleblind like splicing regulator 3 [Source:HGNC Symbol;Acc:HGNC:20564] | 1165 | 0.483 | -0.0143 | No |
| 11 | AKT1 | AKT serine/threonine kinase 1 [Source:HGNC Symbol;Acc:HGNC:391] | 1192 | 0.478 | -0.0106 | No |
| 12 | PRR5L | proline rich 5 like [Source:HGNC Symbol;Acc:HGNC:25878] | 1198 | 0.477 | -0.0052 | No |
| 13 | FTO | FTO alpha-ketoglutarate dependent dioxygenase [Source:HGNC Symbol;Acc:HGNC:24678] | 1314 | 0.456 | -0.0087 | No |
| 14 | PCID2 | PCI domain containing 2 [Source:HGNC Symbol;Acc:HGNC:25653] | 1376 | 0.447 | -0.0081 | No |
| 15 | LSM1 | "LSM1 homolog, mRNA degradation associated [Source:HGNC Symbol;Acc:HGNC:20472]" | 1470 | 0.433 | -0.0102 | No |
| 16 | CARHSP1 | calcium regulated heat stable protein 1 [Source:HGNC Symbol;Acc:HGNC:17150] | 1498 | 0.429 | -0.0072 | No |
| 17 | SNRPA | small nuclear ribonucleoprotein polypeptide A [Source:HGNC Symbol;Acc:HGNC:11151] | 1670 | 0.403 | -0.0158 | No |
| 18 | CNOT7 | CCR4-NOT transcription complex subunit 7 [Source:HGNC Symbol;Acc:HGNC:14101] | 1676 | 0.402 | -0.0113 | No |
| 19 | RBM23 | RNA binding motif protein 23 [Source:HGNC Symbol;Acc:HGNC:20155] | 1678 | 0.402 | -0.0065 | No |
| 20 | NUDT21 | nudix hydrolase 21 [Source:HGNC Symbol;Acc:HGNC:13870] | 1734 | 0.393 | -0.0061 | No |
| 21 | FASTK | Fas activated serine/threonine kinase [Source:HGNC Symbol;Acc:HGNC:24676] | 1834 | 0.380 | -0.0093 | No |
| 22 | TRAF3IP2 | TRAF3 interacting protein 2 [Source:HGNC Symbol;Acc:HGNC:1343] | 1939 | 0.368 | -0.0130 | No |
| 23 | EXOSC7 | exosome component 7 [Source:HGNC Symbol;Acc:HGNC:28112] | 1969 | 0.364 | -0.0109 | No |
| 24 | RBM4 | RNA binding motif protein 4 [Source:HGNC Symbol;Acc:HGNC:9901] | 1970 | 0.364 | -0.0065 | No |
| 25 | PRKCD | protein kinase C delta [Source:HGNC Symbol;Acc:HGNC:9399] | 2237 | 0.335 | -0.0234 | No |
| 26 | MAPK14 | mitogen-activated protein kinase 14 [Source:HGNC Symbol;Acc:HGNC:6876] | 2352 | 0.320 | -0.0285 | No |
| 27 | MYEF2 | myelin expression factor 2 [Source:HGNC Symbol;Acc:HGNC:17940] | 2465 | 0.307 | -0.0337 | No |
| 28 | FASTKD3 | FAST kinase domains 3 [Source:HGNC Symbol;Acc:HGNC:28758] | 2473 | 0.306 | -0.0305 | No |
| 29 | DCPS | "decapping enzyme, scavenger [Source:HGNC Symbol;Acc:HGNC:29812]" | 2570 | 0.295 | -0.0345 | No |
| 30 | ANGEL2 | angel homolog 2 [Source:HGNC Symbol;Acc:HGNC:30534] | 2608 | 0.290 | -0.0339 | No |
| 31 | RBM7 | RNA binding motif protein 7 [Source:HGNC Symbol;Acc:HGNC:9904] | 2620 | 0.288 | -0.0313 | No |
| 32 | DCP2 | decapping mRNA 2 [Source:HGNC Symbol;Acc:HGNC:24452] | 2659 | 0.284 | -0.0308 | No |
| 33 | APOBEC1 | apolipoprotein B mRNA editing enzyme catalytic subunit 1 [Source:HGNC Symbol;Acc:HGNC:604] | 2695 | 0.280 | -0.0302 | No |
| 34 | RAVER2 | "ribonucleoprotein, PTB binding 2 [Source:HGNC Symbol;Acc:HGNC:25577]" | 2956 | 0.254 | -0.0477 | No |
| 35 | FXR1 | FMR1 autosomal homolog 1 [Source:HGNC Symbol;Acc:HGNC:4023] | 3012 | 0.249 | -0.0490 | No |
| 36 | BARD1 | BRCA1 associated RING domain 1 [Source:HGNC Symbol;Acc:HGNC:952] | 3051 | 0.245 | -0.0490 | No |
| 37 | C1QBP | complement C1q binding protein [Source:HGNC Symbol;Acc:HGNC:1243] | 3083 | 0.241 | -0.0485 | No |
| 38 | PARN | poly(A)-specific ribonuclease [Source:HGNC Symbol;Acc:HGNC:8609] | 3100 | 0.240 | -0.0469 | No |
| 39 | TENT5A | terminal nucleotidyltransferase 5A [Source:HGNC Symbol;Acc:HGNC:18345] | 3204 | 0.230 | -0.0522 | No |
| 40 | PUF60 | poly(U) binding splicing factor 60 [Source:HGNC Symbol;Acc:HGNC:17042] | 3237 | 0.227 | -0.0520 | No |
| 41 | SON | SON DNA and RNA binding protein [Source:HGNC Symbol;Acc:HGNC:11183] | 3374 | 0.214 | -0.0601 | No |
| 42 | LARP7 | "La ribonucleoprotein 7, transcriptional regulator [Source:HGNC Symbol;Acc:HGNC:24912]" | 3395 | 0.212 | -0.0592 | No |
| 43 | CIRBP | cold inducible RNA binding protein [Source:HGNC Symbol;Acc:HGNC:1982] | 3441 | 0.207 | -0.0602 | No |
| 44 | RIDA | reactive intermediate imine deaminase A homolog [Source:HGNC Symbol;Acc:HGNC:16897] | 3488 | 0.203 | -0.0614 | No |
| 45 | RBFOX2 | RNA binding fox-1 homolog 2 [Source:HGNC Symbol;Acc:HGNC:9906] | 3523 | 0.200 | -0.0616 | No |
| 46 | MBNL2 | muscleblind like splicing regulator 2 [Source:HGNC Symbol;Acc:HGNC:16746] | 3524 | 0.200 | -0.0592 | No |
| 47 | TENT5C | terminal nucleotidyltransferase 5C [Source:HGNC Symbol;Acc:HGNC:24712] | 3646 | 0.190 | -0.0664 | No |
| 48 | KHDRBS3 | "KH RNA binding domain containing, signal transduction associated 3 [Source:HGNC Symbol;Acc:HGNC:18117]" | 3714 | 0.184 | -0.0695 | No |
| 49 | PRKCA | protein kinase C alpha [Source:HGNC Symbol;Acc:HGNC:9393] | 3728 | 0.183 | -0.0683 | No |
| 50 | KHDRBS1 | "KH RNA binding domain containing, signal transduction associated 1 [Source:HGNC Symbol;Acc:HGNC:18116]" | 3748 | 0.182 | -0.0676 | No |
| 51 | CNOT1 | CCR4-NOT transcription complex subunit 1 [Source:HGNC Symbol;Acc:HGNC:7877] | 3842 | 0.172 | -0.0729 | No |
| 52 | ZC3HAV1 | "zinc finger CCCH-type containing, antiviral 1 [Source:HGNC Symbol;Acc:HGNC:23721]" | 3946 | 0.161 | -0.0790 | No |
| 53 | PTCD2 | pentatricopeptide repeat domain 2 [Source:HGNC Symbol;Acc:HGNC:25734] | 4097 | 0.146 | -0.0891 | No |
| 54 | DIS3 | "DIS3 homolog, exosome endoribonuclease and 3'-5' exoribonuclease [Source:HGNC Symbol;Acc:HGNC:20604]" | 4187 | 0.139 | -0.0945 | No |
| 55 | MAGOH | "mago homolog, exon junction complex subunit [Source:HGNC Symbol;Acc:HGNC:6815]" | 4266 | 0.131 | -0.0990 | No |
| 56 | EXOSC5 | exosome component 5 [Source:HGNC Symbol;Acc:HGNC:24662] | 4295 | 0.128 | -0.0997 | No |
| 57 | YTHDF1 | YTH N6-methyladenosine RNA binding protein 1 [Source:HGNC Symbol;Acc:HGNC:15867] | 4320 | 0.127 | -0.1000 | No |
| 58 | A1CF | APOBEC1 complementation factor [Source:HGNC Symbol;Acc:HGNC:24086] | 4368 | 0.123 | -0.1023 | No |
| 59 | UPF1 | UPF1 RNA helicase and ATPase [Source:HGNC Symbol;Acc:HGNC:9962] | 4434 | 0.118 | -0.1059 | No |
| 60 | KHDRBS2 | "KH RNA binding domain containing, signal transduction associated 2 [Source:HGNC Symbol;Acc:HGNC:18114]" | 4550 | 0.109 | -0.1137 | No |
| 61 | PCBP4 | poly(rC) binding protein 4 [Source:HGNC Symbol;Acc:HGNC:8652] | 4577 | 0.107 | -0.1145 | No |
| 62 | CNOT8 | CCR4-NOT transcription complex subunit 8 [Source:HGNC Symbol;Acc:HGNC:9207] | 4581 | 0.106 | -0.1134 | No |
| 63 | PTBP1 | polypyrimidine tract binding protein 1 [Source:HGNC Symbol;Acc:HGNC:9583] | 4650 | 0.100 | -0.1176 | No |
| 64 | HNRNPD | heterogeneous nuclear ribonucleoprotein D [Source:HGNC Symbol;Acc:HGNC:5036] | 4830 | 0.085 | -0.1307 | No |
| 65 | PAIP1 | poly(A) binding protein interacting protein 1 [Source:HGNC Symbol;Acc:HGNC:16945] | 4851 | 0.083 | -0.1312 | No |
| 66 | ZC3H14 | zinc finger CCCH-type containing 14 [Source:HGNC Symbol;Acc:HGNC:20509] | 4896 | 0.080 | -0.1337 | No |
| 67 | DAZ1 | deleted in azoospermia 1 [Source:HGNC Symbol;Acc:HGNC:2682] | 4920 | 0.078 | -0.1346 | No |
| 68 | E2F1 | E2F transcription factor 1 [Source:HGNC Symbol;Acc:HGNC:3113] | 5137 | 0.061 | -0.1509 | No |
| 69 | SCGB1A1 | secretoglobin family 1A member 1 [Source:HGNC Symbol;Acc:HGNC:12523] | 5235 | 0.053 | -0.1579 | No |
| 70 | PKP1 | plakophilin 1 [Source:HGNC Symbol;Acc:HGNC:9023] | 5248 | 0.052 | -0.1582 | No |
| 71 | EIF4ENIF1 | eukaryotic translation initiation factor 4E nuclear import factor 1 [Source:HGNC Symbol;Acc:HGNC:16687] | 5330 | 0.045 | -0.1641 | No |
| 72 | SNW1 | SNW domain containing 1 [Source:HGNC Symbol;Acc:HGNC:16696] | 5472 | 0.035 | -0.1748 | No |
| 73 | HNRNPA0 | heterogeneous nuclear ribonucleoprotein A0 [Source:HGNC Symbol;Acc:HGNC:5030] | 5485 | 0.033 | -0.1753 | No |
| 74 | PKP3 | plakophilin 3 [Source:HGNC Symbol;Acc:HGNC:9025] | 5510 | 0.032 | -0.1769 | No |
| 75 | CDC73 | cell division cycle 73 [Source:HGNC Symbol;Acc:HGNC:16783] | 5604 | 0.023 | -0.1839 | No |
| 76 | CNOT2 | CCR4-NOT transcription complex subunit 2 [Source:HGNC Symbol;Acc:HGNC:7878] | 5740 | 0.012 | -0.1944 | No |
| 77 | AHCYL1 | adenosylhomocysteinase like 1 [Source:HGNC Symbol;Acc:HGNC:344] | 5837 | 0.006 | -0.2019 | No |
| 78 | PAF1 | "PAF1 homolog, Paf1/RNA polymerase II complex component [Source:HGNC Symbol;Acc:HGNC:25459]" | 5901 | 0.002 | -0.2069 | No |
| 79 | FASTKD5 | FAST kinase domains 5 [Source:HGNC Symbol;Acc:HGNC:25790] | 5982 | -0.003 | -0.2131 | No |
| 80 | RBM42 | RNA binding motif protein 42 [Source:HGNC Symbol;Acc:HGNC:28117] | 6059 | -0.009 | -0.2190 | No |
| 81 | GIGYF2 | GRB10 interacting GYF protein 2 [Source:HGNC Symbol;Acc:HGNC:11960] | 6123 | -0.014 | -0.2238 | No |
| 82 | RBFOX1 | RNA binding fox-1 homolog 1 [Source:HGNC Symbol;Acc:HGNC:18222] | 6152 | -0.016 | -0.2259 | No |
| 83 | CNOT3 | CCR4-NOT transcription complex subunit 3 [Source:HGNC Symbol;Acc:HGNC:7879] | 6183 | -0.019 | -0.2280 | No |
| 84 | DAZL | deleted in azoospermia like [Source:HGNC Symbol;Acc:HGNC:2685] | 6221 | -0.022 | -0.2306 | No |
| 85 | CTR9 | "CTR9 homolog, Paf1/RNA polymerase II complex component [Source:HGNC Symbol;Acc:HGNC:16850]" | 6430 | -0.037 | -0.2466 | No |
| 86 | CPEB1 | cytoplasmic polyadenylation element binding protein 1 [Source:HGNC Symbol;Acc:HGNC:21744] | 6435 | -0.038 | -0.2465 | No |
| 87 | WTAP | WT1 associated protein [Source:HGNC Symbol;Acc:HGNC:16846] | 6598 | -0.049 | -0.2587 | No |
| 88 | MYD88 | MYD88 innate immune signal transduction adaptor [Source:HGNC Symbol;Acc:HGNC:7562] | 6638 | -0.052 | -0.2611 | No |
| 89 | RBM10 | RNA binding motif protein 10 [Source:HGNC Symbol;Acc:HGNC:9896] | 6647 | -0.053 | -0.2611 | No |
| 90 | RNF40 | ring finger protein 40 [Source:HGNC Symbol;Acc:HGNC:16867] | 6681 | -0.054 | -0.2630 | No |
| 91 | METTL3 | "methyltransferase 3, N6-adenosine-methyltransferase complex catalytic subunit [Source:HGNC Symbol;Acc:HGNC:17563]" | 6685 | -0.055 | -0.2626 | No |
| 92 | SF3B4 | splicing factor 3b subunit 4 [Source:HGNC Symbol;Acc:HGNC:10771] | 6777 | -0.061 | -0.2691 | No |
| 93 | PRDX6 | peroxiredoxin 6 [Source:HGNC Symbol;Acc:HGNC:16753] | 6783 | -0.061 | -0.2687 | No |
| 94 | RBM15B | RNA binding motif protein 15B [Source:HGNC Symbol;Acc:HGNC:24303] | 6867 | -0.069 | -0.2744 | No |
| 95 | SLC11A1 | solute carrier family 11 member 1 [Source:HGNC Symbol;Acc:HGNC:10907] | 7153 | -0.092 | -0.2958 | No |
| 96 | PUM2 | pumilio RNA binding family member 2 [Source:HGNC Symbol;Acc:HGNC:14958] | 7186 | -0.095 | -0.2972 | No |
| 97 | SMU1 | SMU1 DNA replication regulator and spliceosomal factor [Source:HGNC Symbol;Acc:HGNC:18247] | 7299 | -0.102 | -0.3048 | No |
| 98 | NOVA1 | NOVA alternative splicing regulator 1 [Source:HGNC Symbol;Acc:HGNC:7886] | 7394 | -0.110 | -0.3109 | No |
| 99 | ELAVL1 | ELAV like RNA binding protein 1 [Source:HGNC Symbol;Acc:HGNC:3312] | 7426 | -0.113 | -0.3119 | No |
| 100 | HNRNPC | heterogeneous nuclear ribonucleoprotein C [Source:HGNC Symbol;Acc:HGNC:5035] | 7437 | -0.114 | -0.3114 | No |
| 101 | DCP1A | decapping mRNA 1A [Source:HGNC Symbol;Acc:HGNC:18714] | 7525 | -0.121 | -0.3168 | No |
| 102 | EIF4A3 | eukaryotic translation initiation factor 4A3 [Source:HGNC Symbol;Acc:HGNC:18683] | 7646 | -0.130 | -0.3247 | No |
| 103 | MEX3D | mex-3 RNA binding family member D [Source:HGNC Symbol;Acc:HGNC:16734] | 7652 | -0.131 | -0.3235 | No |
| 104 | DYRK1A | dual specificity tyrosine phosphorylation regulated kinase 1A [Source:HGNC Symbol;Acc:HGNC:3091] | 7667 | -0.132 | -0.3230 | No |
| 105 | POLR2D | RNA polymerase II subunit D [Source:HGNC Symbol;Acc:HGNC:9191] | 7726 | -0.136 | -0.3259 | No |
| 106 | FASTKD2 | FAST kinase domains 2 [Source:HGNC Symbol;Acc:HGNC:29160] | 7730 | -0.136 | -0.3245 | No |
| 107 | MYOD1 | myogenic differentiation 1 [Source:HGNC Symbol;Acc:HGNC:7611] | 7839 | -0.143 | -0.3313 | No |
| 108 | TARDBP | TAR DNA binding protein [Source:HGNC Symbol;Acc:HGNC:11571] | 7864 | -0.146 | -0.3314 | No |
| 109 | CPSF6 | cleavage and polyadenylation specific factor 6 [Source:HGNC Symbol;Acc:HGNC:13871] | 7885 | -0.147 | -0.3312 | No |
| 110 | TOB1 | "transducer of ERBB2, 1 [Source:HGNC Symbol;Acc:HGNC:11979]" | 7892 | -0.147 | -0.3299 | No |
| 111 | FMR1 | FMRP translational regulator 1 [Source:HGNC Symbol;Acc:HGNC:3775] | 7944 | -0.151 | -0.3321 | No |
| 112 | CPEB3 | cytoplasmic polyadenylation element binding protein 3 [Source:HGNC Symbol;Acc:HGNC:21746] | 8060 | -0.160 | -0.3392 | No |
| 113 | HNRNPM | heterogeneous nuclear ribonucleoprotein M [Source:HGNC Symbol;Acc:HGNC:5046] | 8127 | -0.165 | -0.3424 | No |
| 114 | RNPS1 | RNA binding protein with serine rich domain 1 [Source:HGNC Symbol;Acc:HGNC:10080] | 8275 | -0.177 | -0.3519 | No |
| 115 | CSDC2 | cold shock domain containing C2 [Source:HGNC Symbol;Acc:HGNC:30359] | 8294 | -0.178 | -0.3512 | No |
| 116 | PDE12 | phosphodiesterase 12 [Source:HGNC Symbol;Acc:HGNC:25386] | 8306 | -0.178 | -0.3499 | No |
| 117 | TRAF2 | TNF receptor associated factor 2 [Source:HGNC Symbol;Acc:HGNC:12032] | 8322 | -0.180 | -0.3489 | No |
| 118 | NCBP2 | nuclear cap binding protein subunit 2 [Source:HGNC Symbol;Acc:HGNC:7659] | 8478 | -0.192 | -0.3588 | No |
| 119 | EXOSC8 | exosome component 8 [Source:HGNC Symbol;Acc:HGNC:17035] | 8554 | -0.198 | -0.3623 | No |
| 120 | HNRNPA2B1 | heterogeneous nuclear ribonucleoprotein A2/B1 [Source:HGNC Symbol;Acc:HGNC:5033] | 8576 | -0.200 | -0.3615 | No |
| 121 | TUT7 | terminal uridylyl transferase 7 [Source:HGNC Symbol;Acc:HGNC:25817] | 8629 | -0.203 | -0.3632 | No |
| 122 | RBM8A | RNA binding motif protein 8A [Source:HGNC Symbol;Acc:HGNC:9905] | 8662 | -0.205 | -0.3632 | No |
| 123 | PABPC4 | poly(A) binding protein cytoplasmic 4 [Source:HGNC Symbol;Acc:HGNC:8557] | 8741 | -0.212 | -0.3668 | No |
| 124 | SUPT6H | "SPT6 homolog, histone chaperone and transcription elongation factor [Source:HGNC Symbol;Acc:HGNC:11470]" | 8752 | -0.213 | -0.3650 | No |
| 125 | APEX1 | apurinic/apyrimidinic endodeoxyribonuclease 1 [Source:HGNC Symbol;Acc:HGNC:587] | 8767 | -0.213 | -0.3635 | No |
| 126 | RBM3 | RNA binding motif protein 3 [Source:HGNC Symbol;Acc:HGNC:9900] | 8859 | -0.222 | -0.3680 | No |
| 127 | HNRNPL | heterogeneous nuclear ribonucleoprotein L [Source:HGNC Symbol;Acc:HGNC:5045] | 8892 | -0.224 | -0.3678 | No |
| 128 | RBMX | RNA binding motif protein X-linked [Source:HGNC Symbol;Acc:HGNC:9910] | 8938 | -0.227 | -0.3686 | No |
| 129 | FASTKD1 | FAST kinase domains 1 [Source:HGNC Symbol;Acc:HGNC:26150] | 8942 | -0.227 | -0.3661 | No |
| 130 | GTPBP1 | GTP binding protein 1 [Source:HGNC Symbol;Acc:HGNC:4669] | 8950 | -0.228 | -0.3639 | No |
| 131 | RBMY1A1 | RNA binding motif protein Y-linked family 1 member A1 [Source:HGNC Symbol;Acc:HGNC:9912] | 9014 | -0.233 | -0.3661 | No |
| 132 | SAMD4A | sterile alpha motif domain containing 4A [Source:HGNC Symbol;Acc:HGNC:23023] | 9027 | -0.234 | -0.3642 | No |
| 133 | ZC3H12A | zinc finger CCCH-type containing 12A [Source:HGNC Symbol;Acc:HGNC:26259] | 9030 | -0.234 | -0.3615 | No |
| 134 | VIP | vasoactive intestinal peptide [Source:HGNC Symbol;Acc:HGNC:12693] | 9070 | -0.237 | -0.3617 | No |
| 135 | HSPA8 | heat shock protein family A (Hsp70) member 8 [Source:HGNC Symbol;Acc:HGNC:5241] | 9140 | -0.243 | -0.3642 | No |
| 136 | NCBP1 | nuclear cap binding protein subunit 1 [Source:HGNC Symbol;Acc:HGNC:7658] | 9149 | -0.244 | -0.3619 | No |
| 137 | SERBP1 | SERPINE1 mRNA binding protein 1 [Source:HGNC Symbol;Acc:HGNC:17860] | 9238 | -0.251 | -0.3658 | No |
| 138 | TRA2B | transformer 2 beta homolog [Source:HGNC Symbol;Acc:HGNC:10781] | 9243 | -0.251 | -0.3630 | No |
| 139 | YTHDF3 | YTH N6-methyladenosine RNA binding protein 3 [Source:HGNC Symbol;Acc:HGNC:26465] | 9549 | -0.278 | -0.3837 | No |
| 140 | YBX1 | Y-box binding protein 1 [Source:HGNC Symbol;Acc:HGNC:8014] | 9623 | -0.285 | -0.3861 | No |
| 141 | IKBKE | inhibitor of nuclear factor kappa B kinase subunit epsilon [Source:HGNC Symbol;Acc:HGNC:14552] | 9646 | -0.287 | -0.3843 | No |
| 142 | PAPOLA | poly(A) polymerase alpha [Source:HGNC Symbol;Acc:HGNC:14981] | 9786 | -0.300 | -0.3916 | No |
| 143 | TBRG4 | transforming growth factor beta regulator 4 [Source:HGNC Symbol;Acc:HGNC:17443] | 9818 | -0.302 | -0.3904 | No |
| 144 | HNRNPU | heterogeneous nuclear ribonucleoprotein U [Source:HGNC Symbol;Acc:HGNC:5048] | 9847 | -0.306 | -0.3889 | No |
| 145 | RNASEL | ribonuclease L [Source:HGNC Symbol;Acc:HGNC:10050] | 9965 | -0.317 | -0.3943 | No |
| 146 | TENT4A | terminal nucleotidyltransferase 4A [Source:HGNC Symbol;Acc:HGNC:16705] | 10003 | -0.322 | -0.3933 | No |
| 147 | TRAF5 | TNF receptor associated factor 5 [Source:HGNC Symbol;Acc:HGNC:12035] | 10085 | -0.329 | -0.3957 | No |
| 148 | PUM1 | pumilio RNA binding family member 1 [Source:HGNC Symbol;Acc:HGNC:14957] | 10145 | -0.335 | -0.3963 | No |
| 149 | SRSF3 | serine and arginine rich splicing factor 3 [Source:HGNC Symbol;Acc:HGNC:10785] | 10280 | -0.349 | -0.4027 | No |
| 150 | TUT4 | terminal uridylyl transferase 4 [Source:HGNC Symbol;Acc:HGNC:28981] | 10377 | -0.359 | -0.4059 | Yes |
| 151 | ELAVL4 | ELAV like RNA binding protein 4 [Source:HGNC Symbol;Acc:HGNC:3315] | 10412 | -0.362 | -0.4042 | Yes |
| 152 | GDNF | glial cell derived neurotrophic factor [Source:HGNC Symbol;Acc:HGNC:4232] | 10475 | -0.370 | -0.4046 | Yes |
| 153 | RBM15 | RNA binding motif protein 15 [Source:HGNC Symbol;Acc:HGNC:14959] | 10499 | -0.373 | -0.4019 | Yes |
| 154 | SRSF2 | serine and arginine rich splicing factor 2 [Source:HGNC Symbol;Acc:HGNC:10783] | 10507 | -0.374 | -0.3979 | Yes |
| 155 | ZFP36L2 | ZFP36 ring finger protein like 2 [Source:HGNC Symbol;Acc:HGNC:1108] | 10509 | -0.374 | -0.3935 | Yes |
| 156 | SRPK2 | SRSF protein kinase 2 [Source:HGNC Symbol;Acc:HGNC:11306] | 10522 | -0.376 | -0.3898 | Yes |
| 157 | MBNL1 | muscleblind like splicing regulator 1 [Source:HGNC Symbol;Acc:HGNC:6923] | 10557 | -0.379 | -0.3879 | Yes |
| 158 | CSDE1 | cold shock domain containing E1 [Source:HGNC Symbol;Acc:HGNC:29905] | 10559 | -0.379 | -0.3834 | Yes |
| 159 | EXOSC9 | exosome component 9 [Source:HGNC Symbol;Acc:HGNC:9137] | 10606 | -0.384 | -0.3824 | Yes |
| 160 | DHX9 | DExH-box helicase 9 [Source:HGNC Symbol;Acc:HGNC:2750] | 10627 | -0.386 | -0.3793 | Yes |
| 161 | HNRNPAB | heterogeneous nuclear ribonucleoprotein A/B [Source:HGNC Symbol;Acc:HGNC:5034] | 10632 | -0.386 | -0.3749 | Yes |
| 162 | ROCK2 | Rho associated coiled-coil containing protein kinase 2 [Source:HGNC Symbol;Acc:HGNC:10252] | 10648 | -0.388 | -0.3714 | Yes |
| 163 | SRSF6 | serine and arginine rich splicing factor 6 [Source:HGNC Symbol;Acc:HGNC:10788] | 10659 | -0.389 | -0.3675 | Yes |
| 164 | YTHDC1 | YTH domain containing 1 [Source:HGNC Symbol;Acc:HGNC:30626] | 10687 | -0.391 | -0.3649 | Yes |
| 165 | RBM25 | RNA binding motif protein 25 [Source:HGNC Symbol;Acc:HGNC:23244] | 10800 | -0.405 | -0.3688 | Yes |
| 166 | CELF2 | CUGBP Elav-like family member 2 [Source:HGNC Symbol;Acc:HGNC:2550] | 10832 | -0.409 | -0.3663 | Yes |
| 167 | RBM5 | RNA binding motif protein 5 [Source:HGNC Symbol;Acc:HGNC:9902] | 10849 | -0.410 | -0.3626 | Yes |
| 168 | TIA1 | TIA1 cytotoxic granule associated RNA binding protein [Source:HGNC Symbol;Acc:HGNC:11802] | 10855 | -0.411 | -0.3580 | Yes |
| 169 | NUP98 | nucleoporin 98 and 96 precursor [Source:HGNC Symbol;Acc:HGNC:8068] | 10875 | -0.413 | -0.3545 | Yes |
| 170 | SRSF10 | serine and arginine rich splicing factor 10 [Source:HGNC Symbol;Acc:HGNC:16713] | 10926 | -0.422 | -0.3533 | Yes |
| 171 | SRSF7 | serine and arginine rich splicing factor 7 [Source:HGNC Symbol;Acc:HGNC:10789] | 10930 | -0.422 | -0.3484 | Yes |
| 172 | LARP1 | "La ribonucleoprotein 1, translational regulator [Source:HGNC Symbol;Acc:HGNC:29531]" | 10964 | -0.425 | -0.3459 | Yes |
| 173 | CDK9 | cyclin dependent kinase 9 [Source:HGNC Symbol;Acc:HGNC:1780] | 11071 | -0.441 | -0.3489 | Yes |
| 174 | RC3H2 | ring finger and CCCH-type domains 2 [Source:HGNC Symbol;Acc:HGNC:21461] | 11102 | -0.444 | -0.3459 | Yes |
| 175 | SYNCRIP | synaptotagmin binding cytoplasmic RNA interacting protein [Source:HGNC Symbol;Acc:HGNC:16918] | 11182 | -0.454 | -0.3466 | Yes |
| 176 | HSF1 | heat shock transcription factor 1 [Source:HGNC Symbol;Acc:HGNC:5224] | 11189 | -0.456 | -0.3415 | Yes |
| 177 | CNOT6 | CCR4-NOT transcription complex subunit 6 [Source:HGNC Symbol;Acc:HGNC:14099] | 11212 | -0.459 | -0.3377 | Yes |
| 178 | AGO2 | argonaute RISC catalytic component 2 [Source:HGNC Symbol;Acc:HGNC:3263] | 11246 | -0.463 | -0.3347 | Yes |
| 179 | PRPF19 | pre-mRNA processing factor 19 [Source:HGNC Symbol;Acc:HGNC:17896] | 11269 | -0.467 | -0.3308 | Yes |
| 180 | KHSRP | KH-type splicing regulatory protein [Source:HGNC Symbol;Acc:HGNC:6316] | 11285 | -0.469 | -0.3263 | Yes |
| 181 | SRRM1 | serine and arginine repetitive matrix 1 [Source:HGNC Symbol;Acc:HGNC:16638] | 11302 | -0.471 | -0.3218 | Yes |
| 182 | METTL16 | "methyltransferase 16, N6-methyladenosine [Source:HGNC Symbol;Acc:HGNC:28484]" | 11346 | -0.478 | -0.3194 | Yes |
| 183 | RBM39 | RNA binding motif protein 39 [Source:HGNC Symbol;Acc:HGNC:15923] | 11434 | -0.493 | -0.3203 | Yes |
| 184 | CELF3 | CUGBP Elav-like family member 3 [Source:HGNC Symbol;Acc:HGNC:11967] | 11510 | -0.502 | -0.3201 | Yes |
| 185 | NOCT | nocturnin [Source:HGNC Symbol;Acc:HGNC:14254] | 11566 | -0.510 | -0.3183 | Yes |
| 186 | ZC3H3 | zinc finger CCCH-type containing 3 [Source:HGNC Symbol;Acc:HGNC:28972] | 11571 | -0.511 | -0.3124 | Yes |
| 187 | SLTM | SAFB like transcription modulator [Source:HGNC Symbol;Acc:HGNC:20709] | 11593 | -0.514 | -0.3078 | Yes |
| 188 | ZBTB7A | zinc finger and BTB domain containing 7A [Source:HGNC Symbol;Acc:HGNC:18078] | 11600 | -0.516 | -0.3021 | Yes |
| 189 | CASC3 | CASC3 exon junction complex subunit [Source:HGNC Symbol;Acc:HGNC:17040] | 11630 | -0.522 | -0.2980 | Yes |
| 190 | SF1 | splicing factor 1 [Source:HGNC Symbol;Acc:HGNC:12950] | 11664 | -0.527 | -0.2942 | Yes |
| 191 | LARP4B | La ribonucleoprotein 4B [Source:HGNC Symbol;Acc:HGNC:28987] | 11695 | -0.531 | -0.2902 | Yes |
| 192 | DDX17 | DEAD-box helicase 17 [Source:HGNC Symbol;Acc:HGNC:2740] | 11716 | -0.535 | -0.2852 | Yes |
| 193 | SUPT5H | "SPT5 homolog, DSIF elongation factor subunit [Source:HGNC Symbol;Acc:HGNC:11469]" | 11735 | -0.539 | -0.2801 | Yes |
| 194 | SRPK1 | SRSF protein kinase 1 [Source:HGNC Symbol;Acc:HGNC:11305] | 11889 | -0.569 | -0.2853 | Yes |
| 195 | BTG2 | BTG anti-proliferation factor 2 [Source:HGNC Symbol;Acc:HGNC:1131] | 11917 | -0.576 | -0.2805 | Yes |
| 196 | TNRC6B | trinucleotide repeat containing adaptor 6B [Source:HGNC Symbol;Acc:HGNC:29190] | 11947 | -0.582 | -0.2757 | Yes |
| 197 | CDK11B | cyclin dependent kinase 11B [Source:HGNC Symbol;Acc:HGNC:1729] | 11952 | -0.584 | -0.2689 | Yes |
| 198 | NOVA2 | NOVA alternative splicing regulator 2 [Source:HGNC Symbol;Acc:HGNC:7887] | 12006 | -0.594 | -0.2659 | Yes |
| 199 | YBX3 | Y-box binding protein 3 [Source:HGNC Symbol;Acc:HGNC:2428] | 12024 | -0.599 | -0.2600 | Yes |
| 200 | DHX34 | DExH-box helicase 34 [Source:HGNC Symbol;Acc:HGNC:16719] | 12040 | -0.603 | -0.2538 | Yes |
| 201 | ROCK1 | Rho associated coiled-coil containing protein kinase 1 [Source:HGNC Symbol;Acc:HGNC:10251] | 12046 | -0.605 | -0.2469 | Yes |
| 202 | SNRNP70 | small nuclear ribonucleoprotein U1 subunit 70 [Source:HGNC Symbol;Acc:HGNC:11150] | 12073 | -0.613 | -0.2415 | Yes |
| 203 | MAPKAPK2 | MAPK activated protein kinase 2 [Source:HGNC Symbol;Acc:HGNC:6887] | 12094 | -0.619 | -0.2356 | Yes |
| 204 | EXOSC2 | exosome component 2 [Source:HGNC Symbol;Acc:HGNC:17097] | 12100 | -0.620 | -0.2285 | Yes |
| 205 | FXR2 | FMR1 autosomal homolog 2 [Source:HGNC Symbol;Acc:HGNC:4024] | 12219 | -0.650 | -0.2299 | Yes |
| 206 | U2AF2 | U2 small nuclear RNA auxiliary factor 2 [Source:HGNC Symbol;Acc:HGNC:23156] | 12226 | -0.652 | -0.2225 | Yes |
| 207 | SAMD4B | sterile alpha motif domain containing 4B [Source:HGNC Symbol;Acc:HGNC:25492] | 12247 | -0.659 | -0.2160 | Yes |
| 208 | CPSF7 | cleavage and polyadenylation specific factor 7 [Source:HGNC Symbol;Acc:HGNC:30098] | 12251 | -0.661 | -0.2083 | Yes |
| 209 | NCL | nucleolin [Source:HGNC Symbol;Acc:HGNC:7667] | 12255 | -0.662 | -0.2005 | Yes |
| 210 | DND1 | DND microRNA-mediated repression inhibitor 1 [Source:HGNC Symbol;Acc:HGNC:23799] | 12266 | -0.667 | -0.1932 | Yes |
| 211 | QKI | "QKI, KH domain containing RNA binding [Source:HGNC Symbol;Acc:HGNC:21100]" | 12340 | -0.688 | -0.1906 | Yes |
| 212 | HNRNPK | heterogeneous nuclear ribonucleoprotein K [Source:HGNC Symbol;Acc:HGNC:5044] | 12341 | -0.688 | -0.1822 | Yes |
| 213 | HNRNPA1 | heterogeneous nuclear ribonucleoprotein A1 [Source:HGNC Symbol;Acc:HGNC:5031] | 12356 | -0.693 | -0.1749 | Yes |
| 214 | SAFB2 | scaffold attachment factor B2 [Source:HGNC Symbol;Acc:HGNC:21605] | 12363 | -0.696 | -0.1670 | Yes |
| 215 | REST | RE1 silencing transcription factor [Source:HGNC Symbol;Acc:HGNC:9966] | 12382 | -0.703 | -0.1599 | Yes |
| 216 | CELF1 | CUGBP Elav-like family member 1 [Source:HGNC Symbol;Acc:HGNC:2549] | 12391 | -0.704 | -0.1520 | Yes |
| 217 | SAFB | scaffold attachment factor B [Source:HGNC Symbol;Acc:HGNC:10520] | 12395 | -0.705 | -0.1436 | Yes |
| 218 | SECISBP2 | SECIS binding protein 2 [Source:HGNC Symbol;Acc:HGNC:30972] | 12416 | -0.712 | -0.1366 | Yes |
| 219 | THRAP3 | thyroid hormone receptor associated protein 3 [Source:HGNC Symbol;Acc:HGNC:22964] | 12426 | -0.717 | -0.1286 | Yes |
| 220 | TRA2A | transformer 2 alpha homolog [Source:HGNC Symbol;Acc:HGNC:16645] | 12479 | -0.740 | -0.1237 | Yes |
| 221 | CCNT1 | cyclin T1 [Source:HGNC Symbol;Acc:HGNC:1599] | 12517 | -0.757 | -0.1175 | Yes |
| 222 | NPM1 | nucleophosmin 1 [Source:HGNC Symbol;Acc:HGNC:7910] | 12530 | -0.762 | -0.1092 | Yes |
| 223 | PABPC1 | poly(A) binding protein cytoplasmic 1 [Source:HGNC Symbol;Acc:HGNC:8554] | 12562 | -0.778 | -0.1022 | Yes |
| 224 | JMJD6 | "jumonji domain containing 6, arginine demethylase and lysine hydroxylase [Source:HGNC Symbol;Acc:HGNC:19355]" | 12658 | -0.832 | -0.0996 | Yes |
| 225 | DAZAP1 | DAZ associated protein 1 [Source:HGNC Symbol;Acc:HGNC:2683] | 12686 | -0.854 | -0.0914 | Yes |
| 226 | YTHDF2 | YTH N6-methyladenosine RNA binding protein 2 [Source:HGNC Symbol;Acc:HGNC:31675] | 12694 | -0.860 | -0.0815 | Yes |
| 227 | FUS | FUS RNA binding protein [Source:HGNC Symbol;Acc:HGNC:4010] | 12739 | -0.902 | -0.0741 | Yes |
| 228 | SFSWAP | splicing factor SWAP [Source:HGNC Symbol;Acc:HGNC:10790] | 12747 | -0.908 | -0.0636 | Yes |
| 229 | SRSF4 | serine and arginine rich splicing factor 4 [Source:HGNC Symbol;Acc:HGNC:10786] | 12749 | -0.911 | -0.0526 | Yes |
| 230 | VIM | vimentin [Source:HGNC Symbol;Acc:HGNC:12692] | 12777 | -0.932 | -0.0434 | Yes |
| 231 | TAF15 | TATA-box binding protein associated factor 15 [Source:HGNC Symbol;Acc:HGNC:11547] | 12828 | -1.023 | -0.0350 | Yes |
| 232 | DDX5 | DEAD-box helicase 5 [Source:HGNC Symbol;Acc:HGNC:2746] | 12834 | -1.038 | -0.0228 | Yes |
| 233 | ZFP36 | ZFP36 ring finger protein [Source:HGNC Symbol;Acc:HGNC:12862] | 12846 | -1.052 | -0.0109 | Yes |
| 234 | ZFP36L1 | ZFP36 ring finger protein like 1 [Source:HGNC Symbol;Acc:HGNC:1107] | 12888 | -1.244 | 0.0009 | Yes |
Table: GSEA details [plain text format]

  

Fig 2: GOBP\_REGULATION\_OF\_MRNA\_METABOLIC\_PROCESS      
 Blue-Pink O' Gram in the Space of the Analyzed GeneSet

  

Fig 3: GOBP\_REGULATION\_OF\_MRNA\_METABOLIC\_PROCESS: Random ES distribution      
 Gene set null distribution of ES for **GOBP\_REGULATION\_OF\_MRNA\_METABOLIC\_PROCESS**
